# Diversity and Spatial Distribution of Hydrazine Oxidoreductase (*hzo*) Gene in the Oxygen Minimum Zone Off Costa Rica

**DOI:** 10.1371/journal.pone.0078275

**Published:** 2013-10-31

**Authors:** Liangliang Kong, Hongmei Jing, Takafumi Kataoka, Carolyn Buchwald, Hongbin Liu

**Affiliations:** 1 Division of Life Science, The Hong Kong University of Science and Technology, Clear Water Bay, Kowloon, Hong Kong; 2 Sanya Institute of Deep-Sea Science and Engineering, Chinese Academy of Sciences, Sanya, China; 3 MIT/WHOI Joint Program in Chemical Oceanography, Woods Hole Oceanographic Institution, Woods Hole, Massachusetts, United States of America; National University of Singapore, Singapore

## Abstract

Anaerobic ammonia oxidation (anammox) as an important nitrogen loss pathway has been reported in marine oxygen minimum zones (OMZs), but the community composition and spatial distribution of anammox bacteria in the eastern tropical North Pacific (ETNP) OMZ are poorly determined. In this study, anammox bacterial communities in the OMZ off Costa Rica (CRD-OMZ) were analyzed based on both hydrazine oxidoreductase (*hzo*) genes and their transcripts assigned to cluster 1 and 2. The anammox communities revealed by *hzo* genes and proteins in CRD-OMZ showed a low diversity. Gene quantification results showed that *hzo* gene abundances peaked in the upper OMZs, associated with the peaks of nitrite concentration. Nitrite and oxygen concentrations may therefore colimit the distribution of anammox bacteria in this area. Furthermore, transcriptional activity of anammox bacteria was confirmed by obtaining abundant *hzo* mRNA transcripts through qRT-PCR. A novel *hzo* cluster 2x clade was identified by the phylogenetic analysis and these novel sequences were abundant and widely distributed in this environment. Our study demonstrated that both cluster 1 and 2 anammox bacteria play an active role in the CRD-OMZ, and the cluster 1 abundance and transcriptional activity were higher than cluster 2 in both free-living and particle-attached fractions at both gene and transcriptional levels.

## Introduction

 For a long time, aerobic nitrification and anaerobic denitrification were thought to be the only major pathways for ammonium oxidation and nitrogen (N) loss in the marine N cycle. Anaerobic ammonium oxidation (anammox) as a biologically mediated process, however, was discovered in the wastewater treatment systems in the 1990s [1,2]. More recently, the detection of widely distributed anammox bacteria in natural ecosystems has greatly changed our previous understanding of N sink in the N cycle [3]. It has been estimated that anammox was responsible for 19 to 35% of the N removal in the Golfo Dulce, Costa Rica [4]. More recent studies showed that anammox is the dominant process of N loss, with no denitrification activities detected, in the Benguela upwelling system [5], the Black Sea [6] and the Peruvian oxygen minimum zone [7]. In total, anammox could contribute 30 to 50% to the marine N loss [4,8]. The ubiquitous distribution of anammox bacteria in various oxygen-depleted ecosystems [3], as well as its potentially coupling relationships with denitrification [4], nitrification [6] and dissimilatory nitrate reduction to ammonium (DNRA) [9,10] , further emphasizes the critical role of anammox in the global N budget.

 As autotrophic members in the bacteria order *Planctomycetales* [11], five *Candidatus* genera of anammox bacteria have been identified, including *Candidatus* Brocadia [12], *Candidatus* Kuenenia [13], *Candidatus* Scalindua [14], *Candidatus* Anammoxoglobus [9] and *Candidatus* Jettenia [15]. Previously, only species in the genus *Candidatus* Scalindua were detected in the marine environment, but recently the discovery of *Candidatus* Kuenenia sequences in the deep-sea hydrothermal vents [16] and in the eastern tropical South Pacific water columns [17] further highlights the importance of study on the largely uncharacterized anammox bacterial communities in marine ecosystems. The 16S rRNA gene based primer or probe analysis has greatly extended our view of anammox bacteria from the artificial bioreactors to various natural environments, such as marine/freshwater sediments and water columns [5,18-20]. However, specific 16S rRNA gene primers do not capture the whole diversity of the anammox bacteria and allow assured linkage between bacteria identity and their metabolic capacity [21]. Molecular methods to detect functional gene markers based on the hydrazine oxidoreductase (HZO), the key enzyme catalyzing the oxidation of the important intermediate hydrazine (N_2_H_4_) to dinitrogen (N_2_), were then developed and three *hzo* gene clusters (cluster 1, 2 and 3) have been identified [22]. Recently, estimation of anammox diversity by *hzo* genes has been argued to be complex due to the presence of 8 *hzo* genes in the genome of *Candidatus* Kuenenia stuttgartiensis [21,23]. Another gene encoding hydrazine synthase (*hzs*), which is more specific to anammox bacteria, were therefore selected to analyze anammox bacterial communities in nature [21,24,25]. But there are so far only 164 *hzsA* and 136 *hzsB* sequences available in the NCBI database. Also most *hzs* gene sequences recovered are affiliated into *Kuenenia*, *Jettenia* and *Brocadia* clades [24,25]. Considering that most anammox bacteria in marine waters belong to *Scalindua* clade, we believe that *hzo* genes are still good markers for phylogenetic analysis of anammox bacteria in marine environments. The HZO assigned to cluster 1 were successfully purified from anammox environmental culture KSU-1 and their function in oxidizing N_2_H_4_ has been experimentally confirmed [26]. Very recently, another two HZO proteins, which are now also named as hydrazine dehydrogenase, were purified from the anammox bioreactors with catalytic function being characterized [27]. The HZO assigned to cluster 2 was observed to be able to oxidize N_2_H_4_ to N_2_ but at a much lower rate compared with cluster 1 HZO [27]. The primers targeting *hzo* cluster 1 have been used to detect the functional anammox bacterial community in marine sediments [20,28-30]. However, very few studies, especially for *hzo* gene sequences related to cluster 2, have been reported in marine water columns [31], especially oxygen minimum zones (OMZs) where a substantial proportion of N loss occurs [3].

 Marine OMZs, characterized by stably depleted oxygen concentrations (e.g. < 20 μM), usually occur in intermediate waters of the open ocean [32], including the eastern tropical Pacific and Atlantic oceans, as well as the Arabian Sea in the Indian Ocean [33,34], in association with the upwelling systems [5,35]. Although global OMZs occupy only a small portion (~0.1%) of the ocean, their role in affecting the biogeochemical cycles, especially N cycle, has been recognized [3,32,33]. The release of ammonium and nitrite through incomplete denitrification and deficient dissolved oxygen in the OMZs support the growth of anammox bacteria, making OMZs an important environment for marine N loss. The Costa Rica Dome (CRD), located in the Eastern Tropical North Pacific (ETNP) with a diameter of approximately 300 km, is one of the eastern boundary upwelling systems [36-38]. The permanent upwelling fertilizes the surface layers and the high biomass sinks to subsurface layers consuming the dissolved oxygen by aerobic respiration, therefore generating a stable OMZ between 400 to 700 m [36]. Compared with other well-studied marine oxygen deficient systems, such as the Eastern Tropical South Pacific (ETSP) and Arabian Sea [7,31,39,40], anammox community and activity in the CRD-OMZ (ETNP) are still not well known. Very recently, abundant ladderane fatty acids, the specific biomarker for the anammox bacteria, were detected in CRD-OMZ, indicating the occurrence of the anammox bacteria and their potential significant contribution to the N loss in this unique environment [41]. Therefore, studying anammox bacterial communities in CRD-OMZ may help us to understand their ecological role in the biogeochemical cycling in oxygen-depleted waters. 

 In this study, community compositions of both *hzo* cluster 1 and cluster 2 anammox bacteria in CRD-OMZ were investigated by analysis of clone library. The abundance of *hzo* gene and its transcriptional activity in the two size fractions was quantified by quantitative PCR. Our study provided the first insight into the diversity of anammox bacteria communities in CRD-OMZ, and it was the first attempt to study the *hzo* cluster 2 sequences in the environments as well.

## Materials and Methods

### Sample collection and environmental conditions

**Figure 1:**
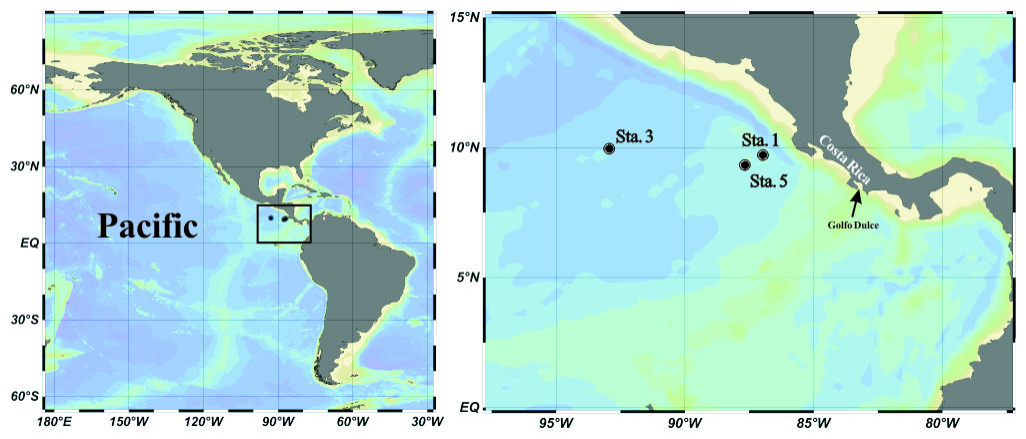
Location of sampling stations in Costa Rica Dome (CRD).

 Sampling was conducted at three stations in the OMZ off Costa Rica coast during the FLUZiE cruise in June and July 2010 (Figure 1). No specific permissions were required for sampling in these locations. All of the three stations are located in the region of the Costa Rica Dome (CRD) with an annual mean thermocline depth of 35 and 40 m (20°C isotherm depth) according to previous study [37]. Seawater samples together with hydrographical data were collected by a conductivity-temperature-depth (CTD) rosette system (Sea-Bird Electronics) with attached Niskin bottles. The dissolved oxygen concentration was measured by a Seabird oxygen sensor attached to the CTD and the range of the OMZ was then determined. For DNA isolation, 1 liter seawater from four to eight depths inside and outside of the OMZs at each station was filtered onto a 2 μm and then a 0.2 μm pore size polycarbonate filter (47 mm, Millipore) with a low vacuum pressure. The same procedure was conducted for RNA sample collection, except that the filters were immersed in RNAlater solution (Ambion) immediately after the filtration. All of the filters were flash frozen and stored at -80°C until further analysis. Nitrite concentrations were measured on board using the Greiss-Ilosvay colorometric method on a UV-VIS spectrophotometer [42]. The ammonium samples were measured on board using a fluorometric method for low concentration samples described in Holmes et al. [43]. The detection limits for nitrite and ammonium concentrations were 0.1 and 0.03 μM, respectively.

### DNA and RNA extraction

 Genomic DNA was extracted from the filters with the PureLink Genomic DNA Kits (Invitrogen, Carlsbad, CA) following the protocol designed with the lysozyme lysis buffer. Briefly, filters were cut into small pieces and incubated in ~200 μl lysozyme digestion buffer (25 mM Tris-HCl, 2.5 mM EDTA, 1% Triton X-100 and 20 mg/ml fresh lysozyme) at 37 °C for 30 min. Proteins were digested from the lysates by incubating with proteinase K (1 mg/ml) and genomic lysis/binding buffer (supplied in the kit) at 55 °C for 30 min. The lysates were then thoroughly mixed with 200 μl absolute ethanol and the genomic DNA was purified by the spin column within the kit. After 2 times washing with buffer, the extracted DNA was eluted into 100 μl TE butter and stored in -80°C. 

 Total RNA was extracted from the Millipore filters with the TRIzol plus RNA purification kit (Invitrogen, Carlsbad, CA). Briefly, the RNAlater immersing the filters was removed before the preparation with TRIzol Reagent, because the residual RNAlater would inhibit the dissociation of nucleoprotein complexes and subsequently reduce the generation of the RNA. The filters were incubated in a 2 ml microcentrifuge tube with 1 ml TRIzol Reagent at room temperature for 5 min. After adding 200 μl chloroform, the tube was shaken by hand for 15s and incubated at room temperature for 3 min. The sample was centrifuged at 12000 g for 15 min at 4 °C and the supernatant was then collected and mixed with an equal volume of 70% ethanol. RNA was then purified with the column following the protocols and finally eluted in 50 μl elution buffer came with the kit. DNA and RNA concentrations were measured on a NanoDrop 1000 Spectrophotometer (Thermo Scientific).

### Phylogenetic analysis of anammox bacterial communities

 Gene fragments of *hzo* cluster 1 were amplified from DNA samples using the primer set of hzoF1 (5'-TGTGCATGGTCAATTGAAAG-3') and hzoR1 (5'-CAACCTCTTCWGCAGGTGCATG-3'), according to the protocol described in Li et al. [29]. Gene fragments of *hzo* cluster 2 from the DNA samples were amplified using the primer set of hzocl2aF1 (5'-GGTTGYCACACAAGGC-3') and hzocl2aR2 (5'-ATATTCACCATGYTTCCAG-3'), according to the protocol described in Schmid et al. [22]. Nuclease-free water was used as negative control in each reaction. To minimize the PCR errors, more than 3 reactions for each sample with various amount of DNA template were pooled together. The positive amplicons of *hzo* cluster 1 (ca. 740 bp) and *hzo* cluster 2 (ca. 790 bp) from each DNA sample were confirmed by electrophoresis on a 1.5% agarose gel and purified using the Gel band purification kit (GE Healthcare, UK) and then ligated into pMD 18-T vector with the cloning kit (TaKaRa, Japan). Correct insertions were checked by direct PCR amplification of randomly selected clones using M13 forward and reverse primers and 15 clones per library were sequenced on a 3730xl DNA Analyzer (Applied Biosystems, Foster City, CA).

 Obtained *hzo* gene sequences were translated into amino acid sequences using Bioedit [44]. The cutoff value of 1% sequence distance was applied to define the operational taxonomic unit (OTU) using the DOTUR [45]. With the representative sequence of each OTU and the top-hit HZO protein sequences from the GenBank by a protein blast (pBLAST) search, a neighbor-joining tree was constructed using MEGA 4.0 [46] with bootstrap analysis with 1000 replicates.

### Quantification of *hzo* cluster 1 and cluster 2 gene sequences

 To quantify anammox *hzo* gene abundances, primers of hzoF1 and hzoR1 for *hzo* cluster 1, and hzocl2aF1 and hzocl2aR1 (5'-TYWACCTGGAACATACCC-3') for *hzo* cluster 2 were applied into quantitative PCR (qPCR). As the primer set hzocl2aF1 and hzocl2aR2 applied for phylogenetic analysis of *hzo* cluster 2 community has a low annealing temperature (48°C), the other reverse primer hzocl2aR1 designed by Schmid et al. [22] was used for the qPCR analysis of *hzo* cluster 2 sequences. This primer sequence has been checked to be identical with the target region of all of the sequences recovered with the other primer set used in this study. Standard curves were determined by analyzing 10-fold serial dilutions of linear plasmids with the target gene inserts and were constructed with linear regression of *C*_T_ values plotted against the initial gene copy number on a log scale. QPCR was performed in triplicates in a final volume of 20 µl reaction with 2 µl of extracted DNA (~2-5 ng) from environmental samples, 1× SYBR Premix Ex Taq (TaKaRa, Japan), 1× ROX reference dye II and 200 nM each of the forward and reverse primer on a 7500 Fast Real-Time PCR system (Applied Biosystems, Foster City, CA), with PCR conditions of 95 °C for 30 s, followed by 40 cycles of 95 °C for 5 s, 56 °C for 20 s for *hzo* cluster 1 (53 °C for *hzo* cluster 2) and 72 °C for 40s. Fluorescence was detected at the PCR extension step at 72 °C. Amplification specificity was determined under a condition of gradual increase in temperature from the annealing temperature to 95 °C in each post-amplification melting curve to confirm that only the target band was generated in each positive reaction. A sample inhibition test was conducted with the addition of 2µl of randomly selected samples to the plasmid reaction and no sample inhibitory effect was detected. The gene copy number was calculated from the *C*_T_ value applied to the regression formula generated from the standard curve.

### cDNA synthesis and quantitative reverse transcription PCR

Before cDNA synthesis, purified total RNA was treated with DNase I to eliminate DNA contamination. Total RNA (up to 200 ng) was then reverse transcribed to cDNA using the SuperScript III first strand cDNA synthesis kit (Invitrogen, Carlsbad, CA). The reaction consisted of 8 μl of RNA treated with DNase I (Amp Grad, Invitrogen), 1 μl of random primers, 1× RT buffer, 5 mM MgCl_2_, 0.5 mM each deoxynucleoside triphosphate (dNTP), 10 mM dithiothreitol, 1 U RNaseOUT and 1 U SuperScript III reverse transcriptase (RT). A parallel reaction without SuperScript III RT was used as RT-PCR negative control (non-RT control). RNA was reverse transcribed at 50 °C for 50 min and the reaction was terminated at 85 °C for 5 min. Residual RNA was removed by addition of 2 U RNase H at 37 °C for 20 min. Two microliter cDNA (1.5 to 8.0 ng) was used for quantitative reverse transcription PCR (qRT-PCR) with the same protocol as mentioned above for qPCR. Both non-RT control and non-template control were always used as a negative control.

### Statistical analyses

 All of the statistical analyses were conducted at the cutoff value of 1% on amino acid level. The *Shannon* and *Simpson* diversity indices, as well as the species richness estimator *Chao* and abundance-based coverage estimator (ACE), were calculated by the DOTUR [45]. The percentage of the community coverage was calculated by the Good’s coverage: Coverage = [1-(n_1_ / N)] ×100, where n_1_ is the number of unique (single-clone) OTUs and the N is the total number of sequences. The Unifrac (http://bmf2.colorado.edu/unifrac/index.psp) weighted principal coordinates analysis (PCoA) was applied to analyze the representative HZO sequences in each OTU to understand the compositional difference of anammox communities among different samples [47]. The significance of the compositional difference among samples was tested by the *P*-test and Unifrac significance test. 

### Nucleotide sequence accession numbers

 All of the obtained sequences were deposited in GenBank with the accession number JN228055 to JN228086 and JN790154 to JN790185.

## Results

### Hydrographic conditions and OMZ determination

**Figure 2:**
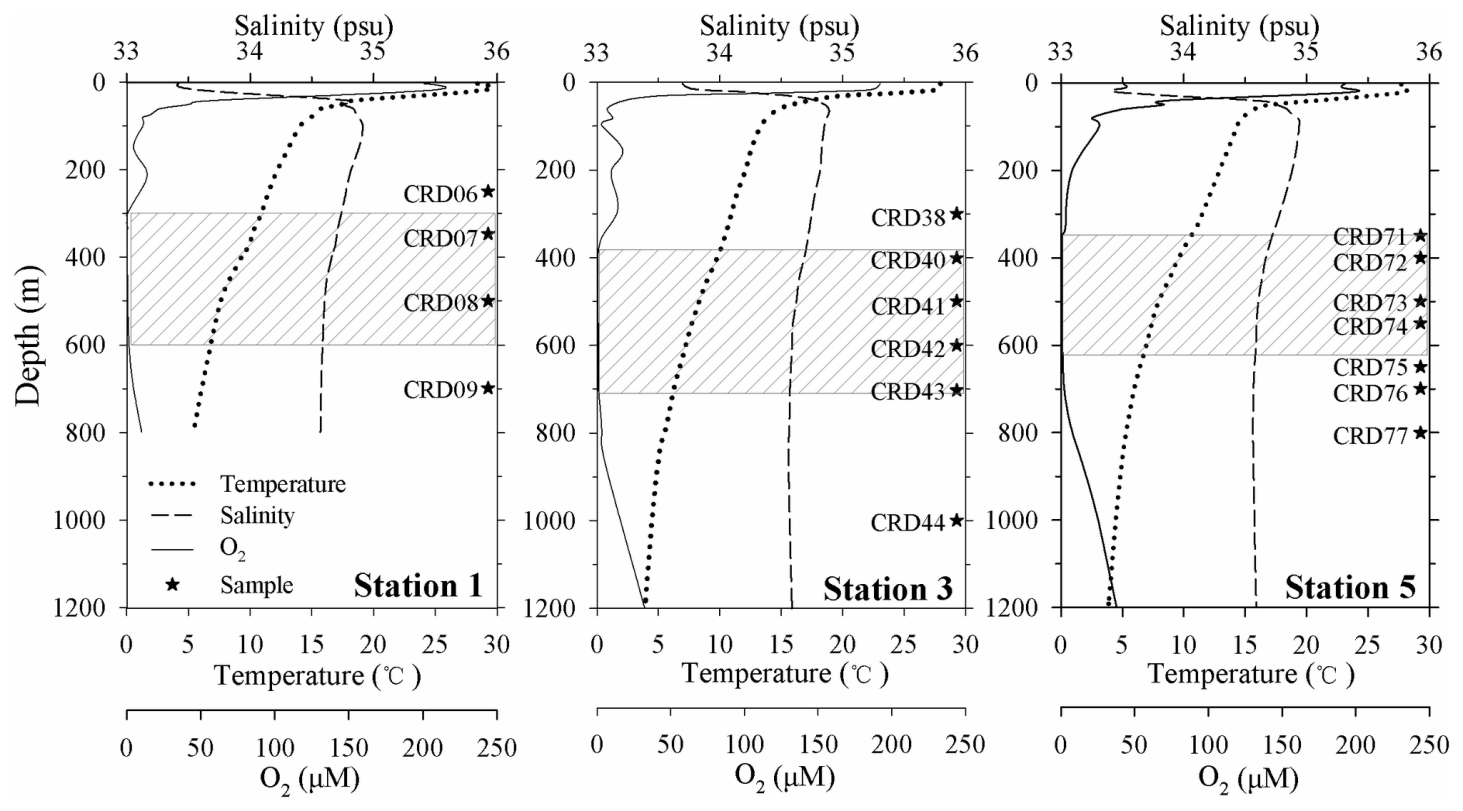
Hydrographic conditions of the sampling stations. Shadow layers indicate the core of the oxygen minimum zones (OMZs) characterized by dissolved oxygen concentration lower than 1.0 μM.

**Figure 3:**
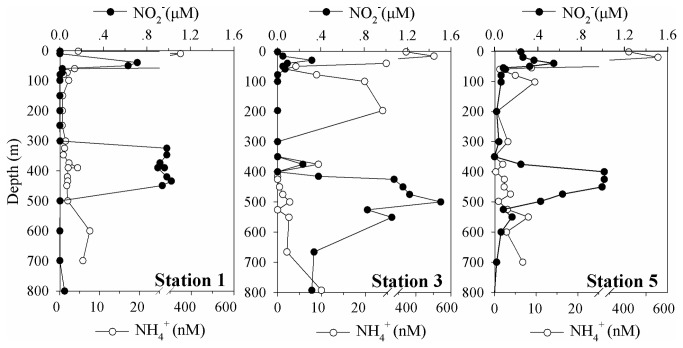
Vertical distributions of nitrite (NO_2_^-^) and ammonium (NH_4_^+^) in the water columns of the sampling stations in Costa Rica Dome (CRD).

 Similar hydrographic characteristics of the water columns were shown at the three stations (Figure 2). Salinity increased and temperature decreased sharply at the depth of 30-40 m, corresponding to the upwelling front, where a stable thermocline was generated. Dissolved oxygen concentration decreased from the surface until a secondary peak appeared at around 200 m depth, and then decreased again to a very low value, where the OMZs of around 300 m thick were formed below 300 m. A subsurface peak of ammonium and nitrite concentrations in the euphotic zone was observed and nitrite concentration reached its maximum of 0.98-1.50 μM within the OMZ. Below the nitrite peak, ammonium began to accumulate with nitrite concentration dropped to the background value (Figure 3). Based on the profiles of dissolved oxygen and nutrients, water layers outside the OMZ (above and below the OMZ) and the core OMZ layer (dissolved oxygen ≤ 1.0 μM) could be clearly defined. Inside the core OMZ layer, the region between the upper OMZ boundary (oxic-suboxic boundary) and the layer of the nitrite peak was defined as the upper OMZ; the region between the layer of nitrite peak and lower OMZ boundary was defined as the lower OMZ.

### *hzo* gene cluster 1 and cluster 2 community diversity and compositions

 In order to reveal the community diversity and compositions of *hzo* cluster 1 and cluster 2 in the CRD-OMZ, 5-6 samples for each gene cluster among different depths (including upper & lower OMZs and the oxic-suboxic boundaries) and different stations were selected for cloning analysis. Overall, 92 *hzo* cluster 1 sequences and 70 *hzo* cluster 2 sequences were obtained from the three stations in CRD-OMZ (Table 1). The recovered *hzo* sequences showed high sequence similarities for cluster 1 (94.1-100%) and cluster 2 (96.7-100%). The deduced amino acid sequences in each cluster were 96.3-100% (cluster 1) and 97.2-100% (cluster 2) identical to one another. At the 1% sequence cutoff on amino acid level, both *hzo* cluster 1 and cluster 2 communities in the whole environment were represented in 9 OTUs, with the community coverage values of 96.7% and 97.1%, respectively. For each specific sample, 13 to 16 sequences were recovered from each clone library. Among them, one to four OTUs were identified with similar diversity indices and species richness estimators (Table 1). The library coverage values ranged from 78.6% to 100%. The weighed PCoA showed that there were no apparent clustering patterns among samples (Figure S1). No significant differences were detected between the HZO communities from different depths and stations by both Unifrac significance test (*P* = 1.000) and *P*-test (*P* = 1.000). 

**Table 1:** Community composition and diversity analysis of oxygen minimum zone (OMZ) samples in Costa Rica Dome (CRD) based on hydrazine oxidoreductase gene deduced amino acid sequences (HZO). Operational taxonomic units (OTUs) were defined at the 1% sequence cutoff value.

Types	Sample^*a*^	Station	Depth (m)	Category^*b*^	No. of clones	No. of OTUs	*Shannon*	*Simpson*	*ACE*	*Chao*	*Coverage* (%)
HZO cluster 1	fCRD07	Sta. 1	350	Upper OMZ	15	4	1.06	0.36	6.2	5.0	86.7
	fCRD41	Sta. 3	500	Upper OMZ	15	3	0.88	0.41	3.6	3.0	93.3
	fCRD71	Sta. 5	350	Upper boundary	15	2	0.69	0.47	2.0	2.0	100
	fCRD72	Sta. 5	400	Upper OMZ	16	4	1.16	0.31	4.6	4.0	93.8
	fCRD73	Sta. 5	500	Lower OMZ	15	3	0.80	0.49	3.8	3.0	93.3
	fCRD75	Sta. 5	650	Lower boundary	16	4	1.28	0.26	4.0	4.0	100
	Total				92	9	1.74	0.21	12.0	12.0	96.7
HZO cluster 2	fCRD07	Sta. 1	350	Upper OMZ	13	1	0	1.00	1.0	1.0	100
	fCRD41	Sta. 3	500	Upper OMZ	14	3	1.01	0.33	3.0	3.0	100
	fCRD72	Sta. 5	400	Upper OMZ	14	4	0.75	0.60	4.7	4.0	78.6
	fCRD73	Sta. 5	500	Lower OMZ	15	3	0.99	0.35	3.0	3.0	100
	fCRD75	Sta. 5	650	Lower boundary	14	3	0.76	0.53	4.0	3.0	92.9
	Total				70	9	1.66	0.24	10.7	9.3	97.1

^*a*^The lowercase letter ‘f’ in the sample names means free living bacteria (size fraction between 0.2-2.0 μm).

^*b*^Category names were defined in the first paragraph in the result.

**Figure 4:**
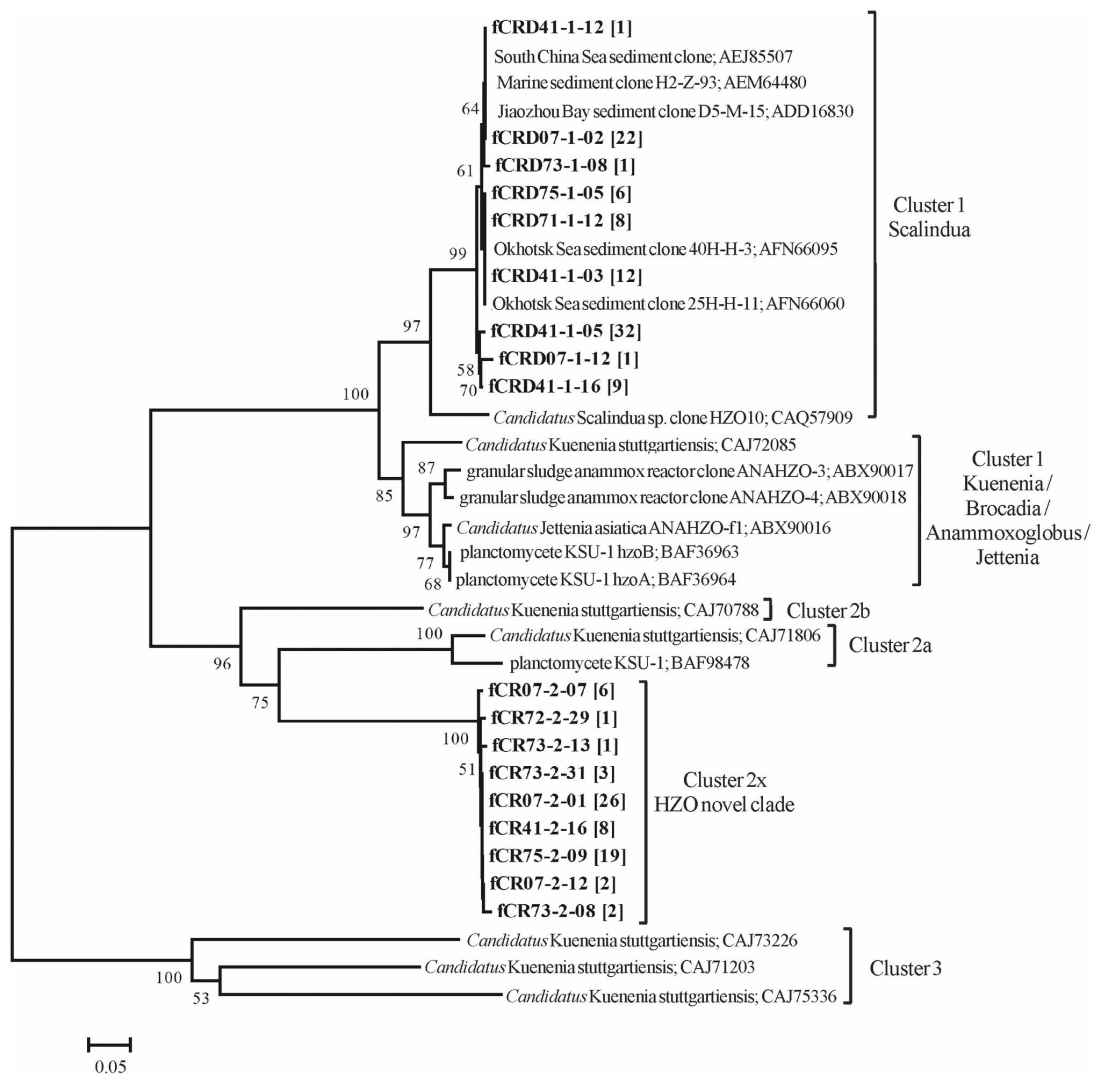
Neighbor-joining phylogenetic tree constructed with anammox bacteria *hzo*-deduced amino acid sequences from the oxygen minimum zone (OMZ) in Costa Rica Dome (CRD). The sequences in bold were obtained in the present study. The sequences were grouped at a 1% cutoff value using DOTUR and only one sequence was selected to represent the operational taxonomic unit (OTU). The number in the brackets followed the sequence name indicated the number of the clones recovered. Bootstrap resampling was performed 1000 times and only its values higher than 50% are shown. The scale bar presents the number of amino acid substitutions per site.

 In contrast to the highly similar community compositions in each gene cluster, lower *hzo* (57.0-59.4%) and HZO (45.2-47.1%) sequence similarities were shared between cluster 1 and cluster 2, and they were separated into two distinct clades in the phylogenetic tree (Figure 4). The topology of phylogenetic tree was supported by different algorithms of maximum parsimony and neighbor joining methods. The obtained HZO cluster 1 sequences were affiliated into the *Candidatus* Scalindua clade. Although the obtained cluster 2 sequences were amplified with the primers previously designed for *hzo* cluster 2a, they were distinct (< 60% of amino acid sequence similarity) from the existing cluster 2a sequences and any other known HZO sequences in the database, therefore we named them *hzo* cluster 2x clade in our study. 

### Quantification of *hzo* gene sequences and their expression

**Figure 5:**
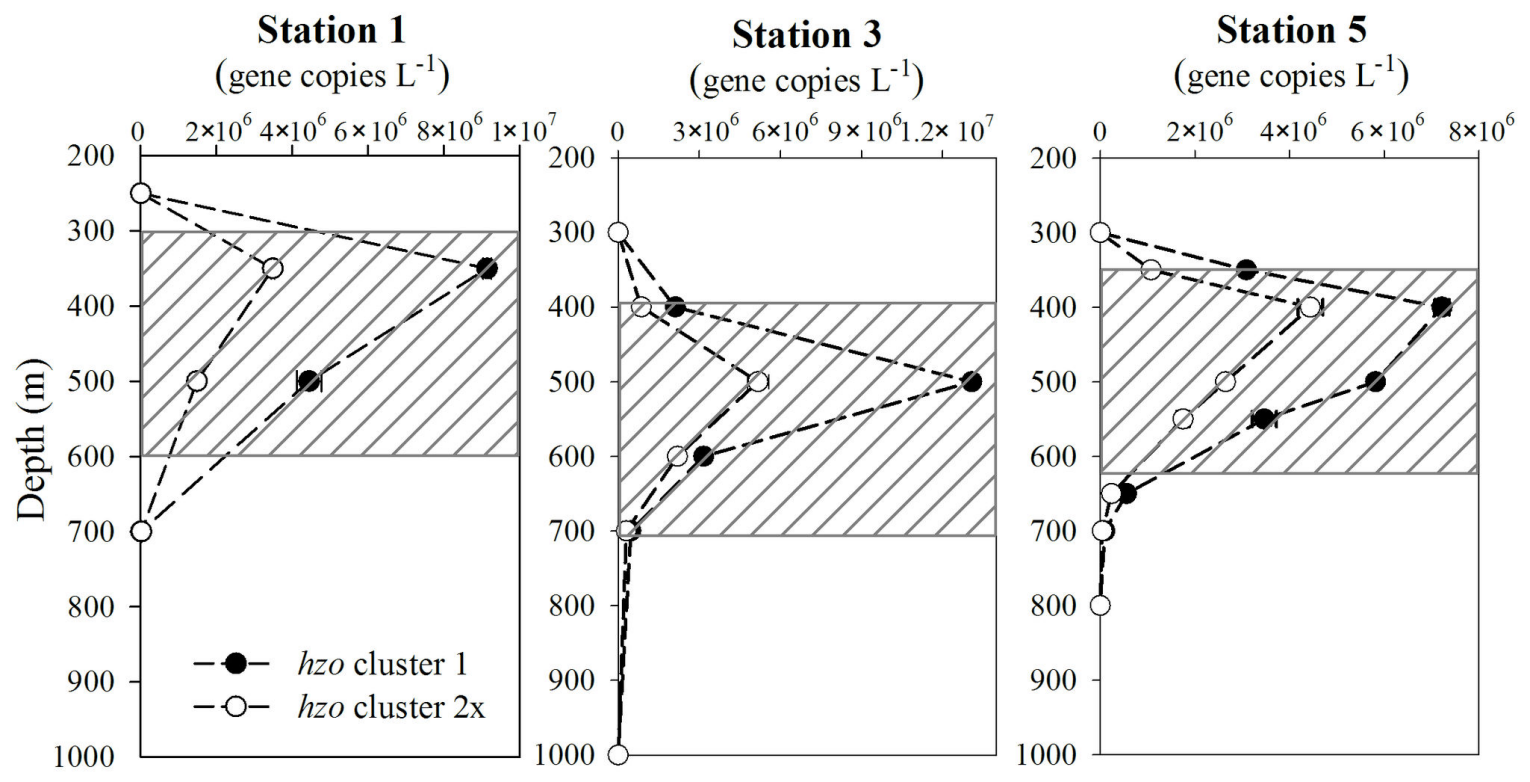
Vertical profiles of *hzo* cluster 1 and cluster 2x gene abundances in the smaller size fraction (0.2-2.0 μm) among different stations in Costa Rica Dome (CRD). Shadow layers show the core of the oxygen minimum zone (OMZ) characterized by dissolved oxygen concentration lower than 1.0 μM.

 To better understand the relationship between the 2 *hzo* gene types and their spatial distribution patterns, the abundances of *hzo* cluster 1 and cluster 2x sequences from two size fractions (> 2.0 μm and 0.2- 2.0 μm) were estimated by qPCR. Two independent standard curves were constructed, with the regression slopes of -3.75 (cluster 1) and -3.56 (cluster 2x), respectively, and high coefficients (*R* ^2^ ≥ 0.99). Both non-template control and non-RT control had *C*_t_ values at least 10 cycles higher than the most diluted plasmid containing the target gene. The post-amplification melting curve analysis clearly showed that there was no target gene contamination in reagents and no DNA contamination in cDNA samples. The abundances of the two gene clusters in the fraction of 0.2- 2.0 μm showed similar distribution patterns at all 3 stations (Figure 5); both of them peaked in the upper OMZs, then decreased with depth and became undetectable below the OMZs. The exact depths of the gene abundance peaks varied among different stations, with peak depths of 350, 500 and 400 m detected at station 1, station 3 and station 5, respectively, corresponding to the peaks of nitrite concentrations at each station (Figures 3 and 5). The maximum gene abundances of the *hzo* cluster 1 ranged from 7.2 × 10^6^ to 1.3 × 10^7^ copies L^-1^ among the 3 stations and the cluster 2x maximum abundances varied from 3.5 × 10^6^ to 5.2 × 10^6^ copies L^-1^. Comparatively, the abundance of *hzo* cluster 1 was 1.4 to 3.0 times higher than that of cluster 2x, except one deep water sample fCRD09 (Table 2). The *hzo* gene abundance of anammox bacteria in the > 2.0 μm fraction, especially for cluster 2x, can only be quantified in a few samples, mainly in the upper OMZs. The gene abundances in the larger size fraction samples were ~100 to 2000 times lower than the smaller size fraction (< 2.0 μm) samples at the same layer of all samples measured (Table 2). The abundance ratios of *hzo* cluster 1: cluster 2x in larger size fraction ranged from ~2 to 10, which were comparable to the ratios of the smaller size fraction samples. 

**Table 2:** Summary of *hzo* gene and transcript abundances from oxygen minimum zone (OMZ) in Costa Rica Dome (CRD).

Station	Sample	Depth	*hzo* gene abundance (< 2.0 μm)			*hzo* gene abundance (> 2.0 μm)			*hzo* transcript^*a*^		
		(m)	(gene copies L^-1^)			(gene copies L^-1^)			(cDNA copies L^-1^)		
			*hzo*cl-1	*hzo*cl-2x	*hzo*cl-1 : *hzo*cl-2x	*hzo*cl-1	*hzo*cl-2x	*hzo*cl-1 : *hzo*cl-2x	*hzo*cl-1	*hzo*cl-2x	*hzo*cl-1 : *hzo*cl-2x
Sta.1	CRD06	250	8.9E+03	5.9E+03	1.5	UD	UD	—			
	CRD07	350	9.2E+06	3.5E+06	2.6	1.8E+04	2.0E+03	8.8	1.6E+06	6.9E+05	2.3
	CRD08	500	4.4E+06	1.5E+06	3.0	5.9E+03	UD	—			
	CRD09	700	1.6E+04	2.7E+04	0.6	UD	UD	—			
Sta.3	CRD38	300	1.8E+03	UD	—	UD	ND	—			
	CRD40	400	2.1E+06	8.6E+05	2.4	6.8E+03	ND	—			
	CRD41	500	1.3E+07	5.2E+06	2.5	2.0E+04	ND	—	2.9E+05	3.2E+05	0.9
	CRD42	600	3.2E+06	2.2E+06	1.4	2.2E+04	ND	—			
	CRD43	700	4.7E+05	3.1E+05	1.5	2.6E+03	ND	—			
	CRD44	1000	UD	UD	—	UD	ND	—			
Sta. 5	CRD69	300	UD	UD	—	UD	UD	—			
	CRD71	350	3.1E+06	1.1E+06	2.9	3.4E+03	8.8E+02	3.9			
	CRD72	400	7.2E+06	4.4E+06	1.6	6.0E+04	5.8E+03	10.3	4.9E+05	1.0E+05	4.8
	CRD73	500	5.8E+06	2.7E+06	2.2	2.0E+03	1.1E+03	1.8			
	CRD74	550	3.5E+06	1.8E+06	2.0	UD	UD	—			
	CRD75	650	5.5E+05	2.4E+05	2.3	UD	UD	—			
	CRD76	700	9.7E+04	4.6E+04	2.1	UD	UD	—			
	CRD77	800	UD	UD	—	UD	UD	—			

^*a*^qRT-PCR analysis was conducted on *hzo* transcripts of free-living anammox bacteria fraction (< 2.0 μm) only at the depth where *hzo* gene peak was detected at each station. UD, under the detection limit; ND, not determined.

In addition, *hzo* gene transcriptional activity was measured in the upper OMZs of the 3 stations where the highest *hzo* gene abundance was recorded. The abundances of *hzo* cluster 1 and cluster 2x cDNA measured by qRT-PCR were 2.9 × 10^5^ to 1.6 × 10^6^ copies L^-1^ and 1.0 × 10^5^ to 6.9 × 10^5^ copies L^-1^, respectively. The cDNA abundances for both gene types were ~5 to 45 times lower than the corresponding gene abundances. The ratios of cluster 1 versus cluster 2x ranged from 0.9 to 4.8.

## Discussion

### Low diversity of anammox HZO community in CRD-OMZ

 Although the sampling size in this study (averaged 15 sequences per sample) for phylogenetic analysis is not very large, the high library coverage values as well as the rarefaction analysis (Figure S2) suggest that most of the HZO community diversity were sufficiently captured and at least the dominant anammox bacteria types were represented. Both the diversity indices and richness estimators indicated low diversity of anammox community in this environment. A low diversity of anammox communities has previously been detected at the 16S rRNA gene level in marine OMZ waters [48,49]. The low diversity observed in this and other studies might be due to the availability and diversity of organic matter exported from the euphotic zones. Bryant et al. [50] suggested that more homogeneous organic matter in marine OMZs reduced the various niches available for microbes and therefore led to declining diversity. Anammox activity has been found to be strongly correlated with the sinking of organic matter in ETSP-OMZ [40]. Organic carbon as an important environmental factor shaping the anammox bacterial community composition has also been reported in marine sediments [28], which retain high amount of organic matter and support higher diversity of anammox communities. 

 The cluster 1 HZO sequences obtained from marine water columns in our study shared 98% to 100% similarity to sequences recovered from marine sediments. But precise identification of our novel cluster 2x sequences seems not possible due to the lack of available HZO cluster 2 sequences. Two reasons might explain the limited number of published cluster 2 sequences. First, the function of cluster 2 HZO is not as well characterized as cluster 1, and their role in anammox process is still not well known [27,51]. Second, there is so far very few well developed primers targeting the cluster 2 [22]. To better understand the taxonomic identity of the anammox bacteria in this environment, 20 anammox bacteria 16S rRNA gene sequences were also recovered from the core OMZs (Figure S3). All of them were closely related to sequences from Arabian Sea and Chilean OMZs [49,52] and affiliated into the *Candidatus* Scalindua *arabica* clade. Previous studies showed that a similar phylogeny was shared between anammox cluster 1 *hzo* and 16S rRNA gene sequences [9,22] , but such a relationship has not been tested for cluster 2 sequences. Extra efforts are required to further identify these novel cluster 2x sequences.

### Oxygen and nitrite co-limiting distribution of anammox bacteria

 The abundance of *hzo* cluster 1 sequences measured in this study is ~100 times lower than that obtained in the Arabian Sea OMZ [31]. However, the ratio of *hzo* cDNA versus *hzo* gene abundances in Arabian Sea is lower than 10^-7^, but the ratio is around 0.02 ~0.2 in our study. This indicated that the *hzo* transcriptional activity in CRD-OMZ is much higher than in the Arabian Sea. The distributions of both types of *hzo* sequences are limited in the core of the OMZ, where oxygen concentration is lower than 1 μM (Figure 4). Associating with the low oxygen content, there is a ~200 m thick water layer with elevated nitrite concentration (> 0.2 μM). The abundances of the two clusters have a very significant correlation with the nitrite concentration in the OMZ (Figure S4; Spearman’s rho correlation: *r* = 0.78, *p* < 0.001 for cluster 1 and *r* = 0.85, *p* < 0.001 for cluster 2x). Previous studies show that oxygen-nitrite co-limit the distribution of anammox bacteria in the Black Sea [5], Arabian Sea [31] and eastern tropical north Pacific [41]. One station (Station 8; 9°00’N, 90°00’W) in Rush et al. [41] is quite close to our station 5 (9°21’N, 87°40’W) in the present study and they also observed an apparent anammox ladderane lipids peak around 400 m associated with a nitrite peak (1.13 μM). Therefore, in this study, nitrite concentration as the substrate for anammox process may also be the limiting factor affecting the distribution of anammox bacteria in the oxygen deficient waters. 

 In the Namibian upwelling system, Woebken and colleagues found that the anammox bacteria *Candidatus* Scalindua spp. were potentially organized into aggregates or attached to particles associated with other bacterial and archaeal partners [49]. In our study, gene quantification of the two size fraction samples (separated by 2.0 μm) may also help us to understand the lifestyle of anammox bacteria in this environment. The sharply more abundant (~100 to 2000 folds, Table 2) *hzo* sequences in the smaller size fraction strongly indicates that more than 99% of anammox bacteria in CRD-OMZ might be in free-living lifestyle. And those *hzo* sequences detected in the larger size fraction might be from cells left on some large particles during filtration.

### Potential source and function of the novel cluster 2x sequences

 To check whether the novel *hzo* cluster 2x sequences are functional or just non-coding gene fragments in the genome, the deduced amino acid sequences were aligned with other known HZO sequences (Figure S5). The novel HZO sequences contain all of the structurally and functionally important amino acid residues conserved in other known HZO sequences, including the active heme-banding motifs C××CH (Heme 5, 6, 7, 8) and the axial ligands His residues (His 2’, 5’, 7’, 8’), as well as the functional amino acids Asp267, His268 and Tyr467, among which the Tyr467 represents the oxidative catalysis as the key function of the enzyme [53]. In addition, highly abundant cluster 2x transcripts were detected, suggesting their potential function in this environment. 

 The metagenomic identification of *hzoA*/*hzoB* gene fragments from the anammox species KSU-1 [26] and eight *hzo* gene fragments in the genome of *Candidatus* Kuenenia stuttgartiensis [22,23] suggested the possibilities of multi-copies of *hzo* gene and coexistence of *hzo* genes from different clusters in one anammox bacterial genome. However, without further evidence, we cannot make a clear conclusion whether or not these novel cluster 2x sequences recovered were from the same species as cluster 1.

 Kartal et al. [27] recently found that one *hzo* cluster 2 sequence in *K. Stuttgartiensis* cells could oxidize hydroxylamine to nitric oxide (NO) at a much higher rate than the N_2_H_4_ oxidation as the key function of HZO. Therefore the cluster 2x sequences detected in this study might also function in the hydroxylamine oxidation. The coupling relationship between anammox and nitrification has been suggested in the Black Sea suboxic zone, where microaerobic nitrification may occur [6]. Also, abundant *amoA* gene, which encodes the alpha subunit of the enzyme Ammonia Monooxygenase for oxidizing ammonium to hydroxylamine as the first step of nitrification, were detected in the OMZs in Arabian Sea [31] and eastern tropical South Pacific [54,55]. However, very few studies reported the hydroxylamine oxidation in marine OMZs. Therefore these novel cluster 2x sequences might be homologous gene encoding hydroxylamine oxidase (HAO) and function as hydroxylamine oxidation to provide nitrite for the anammox process. However, as NO_2_^-^ is at the central position of N cycle to link nitrification, denitrification, anammox and DNRA, its flux and turnover in marine OMZs is highly dynamic and complex. Our study contributes to the first step to study the *hzo* cluster 2 in anammox bacteria; but the function of these novel sequences and their relationship with anammox process requires further studies.

 In summary, in contrast to the ETSP and Arabian Sea OMZs where anammox process has been well studied, our study provided the first insight into the composition and distribution of the anammox bacterial communities in the CRD-OMZ in ETNP. The anammox bacterial communities in this study showed relatively low diversity and their distribution was always limited in the core of the OMZ and correlated well with nitrite concentration. The novel cluster 2x sequences were found quite abundant and they co-occurred with cluster 1 sequences in CRD-OMZ, but their source and function in this special environment remains unclear. 

## Supporting Information

Figure S1 **Unifrac weighted PCoA of HZO community composition using the HZO cluster 1 and cluster 2 amino acid sequences.**
(DOC)

Figure S2 **Rarefaction analysis of the six HZO cluster 1 and five HZO cluster 2 clone libraries.**
(DOC)

Figure S3 **Neighbor-joining phylogenetic tree constructed with anammox bacteria 16S rRNA gene sequences from the oxygen minimum zone (OMZ) in Costa Rica Dome (CRD).**
(DOC)

Figure S4 **Spearman's rho correlation of the *hzo* cluster 1 and cluster 2x sequences with nitrite concentration.**
(DOC)

Figure S5 **Alignment of selected HZO cluster 1 and cluster 2x gene deduced amino acid sequences from this study and the representative HZO sequences from the Genbank.**
(DOC)

## References

[1] Mulder A, van de Graaf AA, Robertson LA, Kuenen JG (1995) Anaerobic ammonium oxidation discovered in a denitrifying fluidized bed reactor. FEMS Microbiol Ecol 16: 177-183. doi:10.1111/j.1574-6941.1995.tb00281.x.

[2] van de Graaf AA, Mulder A, de Bruijn P, Jetten MSM, Robertson LA et al. (1995) Anaerobic oxidation of ammonium is a biologically mediated process. Appl Environ Microbiol 61: 1246-1251. PubMed: 7747947. 7747947 10.1128/aem.61.4.1246-1251.1995 PMC167380

[3] Francis CA, Beman JM, Kuypers MM (2007) New processes and players in the nitrogen cycle: the microbial ecology of anaerobic and archaeal ammonia oxidation. ISME J 1: 19-27. doi:10.1038/ismej.2007.8. PubMed: 18043610. 18043610

[4] Dalsgaard T, Canfield DE, Petersen J, Thamdrup B, Acuña-González J (2003) N_2_ production by the anammox reaction in the anoxic water column of Golfo Dulce, Costa Rica. Nature 422: 606-608. doi:10.1038/nature01526. PubMed: 12686998. 12686998

[5] Kuypers MM, Lavik G, Woebken D, Schmid M, Fuchs BM et al. (2005) Massive nitrogen loss from the Benguela upwelling system through anaerobic ammonium oxidation. Proc Natl Acad Sci U_S_A 102: 6478-6483. doi:10.1073/pnas.0502088102. PubMed: 15843458. 15843458 PMC556276

[6] Lam P, Jensen MM, Lavik G, McGinnis DF, Müller B et al. (2007) Linking crenarchaeal and bacterial nitrification to anammox in the Black Sea. Proc Natl Acad Sci U_S_A 104: 7104-7109. doi:10.1073/pnas.0611081104. PubMed: 17420469. 17420469 PMC1849958

[7] Hamersley MR, Lavik G, Woebken D, Rattray JE, Lam P (2007) Anaerobic ammonium oxidation in the Peruvian oxygen minimum zone. Limnol Oceanogr 52: 923-933. doi:10.4319/lo.2007.52.3.0923.

[8] Devol AH (2003) Nitrogen cycle: Solution to a marine mystery. Nature 422: 575-576. doi:10.1038/422575a. PubMed: 12686985. 12686985

[9] Kartal B, Kuypers MM, Lavik G, Schalk J, Op den Camp HJ et al. (2007) Anammox bacteria disguised as denitrifiers: nitrate reduction to dinitrogen gas via nitrite and ammonium. Environ Microbiol 9: 635-642. doi:10.1111/j.1462-2920.2006.01183.x. PubMed: 17298364. 17298364

[10] Jensen MM, Lam P, Revsbech NP, Nagel B, Gaye B et al. (2011) Intensive nitrogen loss over the Omani Shelf due to anammox coupled with dissimilatory nitrite reduction to ammonium. ISME J 5: 1660-1670. doi:10.1038/ismej.2011.44. PubMed: 21509044. 21509044 PMC3176517

[11] Strous M, Fuerst JA, Kramer EH, Logemann S, Muyzer G et al. (1999) Missing lithotroph identified as new planctomycete. Nature 400: 446-449. doi:10.1038/22749. PubMed: 10440372. 10440372

[12] Strous M, Heijnen JJ, Kuenen JG, Jetten MSM (1998) The sequencing batch reactor as a powerful tool for the study of slowly growing anaerobic ammonium-oxidizing microorganisms. Appl Microbiol Biotechnol 50: 589-596. doi:10.1007/s002530051340.

[13] Schmid M, Twachtmann U, Klein M, Strous M, Juretschko S et al. (2000) Molecular Evidence for Genus Level Diversity of Bacteria Capable of Catalyzing Anaerobic Ammonium Oxidation. Syst Appl Microbiol 23: 93-106. doi:10.1016/S0723-2020(00)80050-8. PubMed: 10879983. 10879983

[14] Schmid M, Walsh K, Webb R, Rijpstra WI, van de Pas-Schoonen K et al. (2003) *Candidatus* "Scalindua brodae", sp. nov., *Candidatus* "Scalindua wagneri", sp. nov., two new species of anaerobic ammonium oxidizing bacteria. Syst Appl Microbiol 26: 529-538. doi:10.1078/072320203770865837. PubMed: 14666981. 14666981

[15] Quan ZX, Rhee SK, Zuo JE, Yang Y, Bae JW et al. (2008) Diversity of ammonium-oxidizing bacteria in a granular sludge anaerobic ammonium-oxidizing (anammox) reactor. Environ Microbiol 10: 3130-3139. doi:10.1111/j.1462-2920.2008.01642.x. PubMed: 18479446. 18479446

[16] Byrne N, Strous M, Crépeau V, Kartal B, Birrien JL et al. (2009) Presence and activity of anaerobic ammonium-oxidizing bacteria at deep-sea hydrothermal vents. ISME J 3: 117-123. doi:10.1038/ismej.2008.72. PubMed: 18670398. 18670398

[17] Stewart FJ, Ulloa O, DeLong EF (2012) Microbial metatranscriptomics in a permanent marine oxygen minimum zone. Environ Microbiol 14: 23-40. doi:10.1111/j.1462-2920.2010.02400.x. PubMed: 21210935. 21210935

[18] Schubert CJ, Durisch-Kaiser E, Wehrli B, Thamdrup B, Lam P et al. (2006) Anaerobic ammonium oxidation in a tropical freshwater system (Lake Tanganyika). Environ Microbiol 8: 1857-1863. doi:10.1111/j.1462-2920.2006.01074.x. PubMed: 16958766. 16958766

[19] Dale OR, Tobias CR, Song B (2009) Biogeographical distribution of diverse anaerobic ammonium oxidizing (anammox) bacteria in Cape Fear River Estuary. Environ Microbiol 11: 1194-1207. doi:10.1111/j.1462-2920.2008.01850.x. PubMed: 19161435. 19161435

[20] Dang H, Zhou H, Zhang Z, Yu Z, Hua E et al. (2013) Molecular detection of *Candidatus* Scalindua pacifica and environmental responses of sediment anammox bacterial community in the Bohai Sea, China. PLOS ONE 8: e61330. doi:10.1371/journal.pone.0061330. PubMed: 23577216. 23577216 PMC3620062

[21] Harhangi HR, Le Roy M, van Alen T, Hu BL, Groen J et al. (2012) Hydrazine synthase, a unique phylomarker with which to study the presence and biodiversity of anammox bacteria. Appl Environ Microbiol 78: 752-758. doi:10.1128/AEM.07113-11. PubMed: 22138989. 22138989 PMC3264106

[22] Schmid MC, Hooper AB, Klotz MG, Woebken D, Lam P et al. (2008) Environmental detection of octahaem cytochrome c hydroxylamine/hydrazine oxidoreductase genes of aerobic and anaerobic ammonium-oxidizing bacteria. Environ Microbiol 10: 3140-3149. doi:10.1111/j.1462-2920.2008.01732.x. PubMed: 18973625. 18973625

[23] Strous M, Pelletier E, Mangenot S, Rattei T, Lehner A et al. (2006) Deciphering the evolution and metabolism of an anammox bacterium from a community genome. Nature 440: 790-794. doi:10.1038/nature04647. PubMed: 16598256. 16598256

[24] Wang Y, Zhu G, Harhangi HR, Zhu B, Jetten MS et al. (2012) Co-occurrence and distribution of nitrite-dependent anaerobic ammonium and methane-oxidizing bacteria in a paddy soil. FEMS Microbiol Lett 336: 79-88. doi:10.1111/j.1574-6968.2012.02654.x. PubMed: 22889245. 22889245

[25] Wang S, Zhu G, Peng Y, Jetten MSM, Yin C (2012) Anammox Bacterial Abundance, Activity, and Contribution in Riparian Sediments of the Pearl River Estuary. Environ Sci Technol 46: 8834-8842. doi:10.1021/es3017446. PubMed: 22816681. 22816681

[26] Shimamura M, Nishiyama T, Shigetomo H, Toyomoto T, Kawahara Y et al. (2007) Isolation of a multiheme protein with features of a hydrazine-oxidizing enzyme from an anaerobic ammonium-oxidizing enrichment culture. Appl Environ Microbiol 73: 1065-1072. doi:10.1128/AEM.01978-06. PubMed: 17172456. 17172456 PMC1828659

[27] Kartal B, Maalcke WJ, de Almeida NM, Cirpus I, Gloerich J et al. (2011) Molecular mechanism of anaerobic ammonium oxidation. Nature 479: 127-130. doi:10.1038/nature10453. PubMed: 21964329. 21964329

[28] Dang H, Chen R, Wang L, Guo L, Chen P et al. (2010) Environmental factors shape sediment anammox bacterial communities in hypernutrified Jiaozhou Bay, China. Appl Environ Microbiol 76: 7036-7047. doi:10.1128/AEM.01264-10. PubMed: 20833786. 20833786 PMC2976235

[29] Li M, Hong Y, Klotz MG, Gu JD (2010) A comparison of primer sets for detecting 16S rRNA and hydrazine oxidoreductase genes of anaerobic ammonium-oxidizing bacteria in marine sediments. Appl Microbiol Biotechnol 86: 781-790. doi:10.1007/s00253-009-2361-5. PubMed: 20107988. 20107988

[30] Hong YG, Yin B, Zheng TL (2011) Diversity and abundance of anammox bacterial community in the deep-ocean surface sediment from equatorial Pacific. Appl Microbiol Biotechnol 89: 1233-1241. doi:10.1007/s00253-010-2925-4. PubMed: 20949269. 20949269

[31] Pitcher A, Villanueva L, Hopmans EC, Schouten S, Reichart GJ et al. (2011) Niche segregation of ammonia-oxidizing archaea and anammox bacteria in the Arabian Sea oxygen minimum zone. ISME J 5: 1896-1904. doi:10.1038/ismej.2011.60. PubMed: 21593795. 21593795 PMC3223301

[32] Ulloa O, Pantoja S (2009) The oxygen minimum zone of the eastern South Pacific. Deep Sea Res II 56: 987-991. doi:10.1016/j.dsr2.2008.12.004.

[33] Paulmier A, Ruiz-Pino D (2009) Oxygen minimum zones (OMZs) in the modern ocean. Prog Oceanogr 80: 113-128. doi:10.1016/j.pocean.2008.08.001.

[34] Karstensen J, Stramma L, Visbeck M (2008) Oxygen minimum zones in the eastern tropical Atlantic and Pacific oceans. Prog Oceanogr 77: 331-350. doi:10.1016/j.pocean.2007.05.009.

[35] Woebken D, Fuchs BM, Kuypers MM, Amann R (2007) Potential interactions of particle-associated anammox bacteria with bacterial and archaeal partners in the Namibian upwelling system. Appl Environ Microbiol 73: 4648-4657. doi:10.1128/AEM.02774-06. PubMed: 17526789. 17526789 PMC1932835

[36] Wyrtki K (1984) Upwelling in the costa rica dome. Fish Bull 63: 355-372.

[37] Fiedler PC (2002) The annual cycle and biological effects of the Costa Rica Dome. Deep Sea Res I 49: 321-338. doi:10.1016/S0967-0637(01)00057-7.

[38] Helly JJ, Levin LA (2004) Global distribution of naturally occurring marine hypoxia on continental margins. Deep Sea Res I 51: 1159-1168. doi:10.1016/j.dsr.2004.03.009.

[39] Lam P, Lavik G, Jensen MM, van de Vossenberg J, Schmid M et al. (2009) Revising the nitrogen cycle in the Peruvian oxygen minimum zone. Proc Natl Acad Sci U_S_A 106: 4752-4757. doi:10.1073/pnas.0812444106. PubMed: 19255441. 19255441 PMC2649953

[40] Kalvelage T, Lavik G, Lam P, Contreras S, Arteaga L et al. (2013) Nitrogen cycling driven by organic matter export in the South Pacific oxygen minimum zone. Nature Geosci 6: 228-234.

[41] Rush D, Wakeham SG, Hopmans EC, Schouten S, Sinninghe Damsté JS (2012) Biomarker evidence for anammox in the oxygen minimum zone of the Eastern Tropical North Pacific. Org Geochem 53: 80-87. doi:10.1016/j.orggeochem.2012.02.005.

[42] Strickland JDH, Parsons TR (1972) A practical handbook of seawater analysis. Bull Fish Res Bd Can 167: 1-310.

[43] Holmes RM, Aminot A, Kérouel R, Hooker BA, Peterson BJ (1999) A simple and precise method for measuring ammonium in marine and freshwater ecosystems. Can J Fish Aquat Sci 56: 1801-1808. doi:10.1139/f99-128.

[44] Hall TA (1999) BioEdit: a user-friendly biological sequence alignment editor and analysis program for Windows 95/98/NT. Nuc Acids Symp SE 41: 95-98.

[45] Schloss PD, Handelsman J (2005) Introducing DOTUR, a computer program for defining operational taxonomic units and estimating species richness. Appl Environ Microbiol 71: 1501-1506. doi:10.1128/AEM.71.3.1501-1506.2005. PubMed: 15746353. 15746353 PMC1065144

[46] Tamura K, Dudley J, Nei M, Kumar S (2007) MEGA4: Molecular Evolutionary Genetics Analysis (MEGA) software version 4.0. Mol Biol Evol 24: 1596-1599. doi:10.1093/molbev/msm092. PubMed: 17488738. 17488738

[47] Lozupone C, Hamady M, Knight R (2006) UniFrac--an online tool for comparing microbial community diversity in a phylogenetic context. BMC Bioinformatics 7: 371. doi:10.1186/1471-2105-7-371. PubMed: 16893466. 16893466 PMC1564154

[48] Schmid MC, Risgaard-Petersen N, van de Vossenberg J, Kuypers MM, Lavik G et al. (2007) Anaerobic ammonium-oxidizing bacteria in marine environments: widespread occurrence but low diversity. Environ Microbiol 9: 1476-1484. doi:10.1111/j.1462-2920.2007.01266.x. PubMed: 17504485. 17504485

[49] Woebken D, Lam P, Kuypers MM, Naqvi SW, Kartal B et al. (2008) A microdiversity study of anammox bacteria reveals a novel Candidatus Scalindua phylotype in marine oxygen minimum zones. Environ Microbiol 10: 3106-3119. doi:10.1111/j.1462-2920.2008.01640.x. PubMed: 18510553. 18510553

[50] Bryant JA, Stewart FJ, Eppley JM, DeLong EF (2012) Microbial community phylogenetic and trait diversity declines with depth in a marine oxygen minimum zone. Ecology 93: 1659–1673. doi:10.1890/11-1204.1. PubMed: 22919912. 22919912

[51] Shimamura M, Nishiyama T, Shinya K, Kawahara Y, Furukawa K et al. (2008) Another multiheme protein, hydroxylamine oxidoreductase, abundantly produced in an anammox bacterium besides the hydrazine-oxidizing enzyme. J Biosci Bioeng 105: 243-248. doi:10.1263/jbb.105.243. PubMed: 18397776. 18397776

[52] Galán A, Molina V, Thamdrup B, Woebken D, Lavik G et al. (2009) Anammox bacteria and the anaerobic oxidation of ammonium in the oxygen minimum zone off northern Chile. Deep Sea Res II 56: 1021-1031. doi:10.1016/j.dsr2.2008.09.016. 10.1016/j.dsr2.2008.09.016

[53] Klotz MG, Schmid MC, Strous M, op den Camp HJ, Jetten MS et al. (2008) Evolution of an octahaem cytochrome c protein family that is key to aerobic and anaerobic ammonia oxidation by bacteria. Environ Microbiol 10: 3150-3163. doi:10.1111/j.1462-2920.2008.01733.x. PubMed: 18761666. 18761666

[54] Molina V, Farías L (2009) Aerobic ammonium oxidation in the oxycline and oxygen minimum zone of the eastern tropical South Pacific off northern Chile (^~^20°S). Deep Sea Res II 56: 1032-1041. doi:10.1016/j.dsr2.2008.09.006.

[55] Farías L, Castro-Gonza lez M, Cornejo M, Charpentier J, Faundez J (2009) Denitrification and nitrous oxide cycling within the upper oxycline of the eastern tropical South Pacific oxygen minimum zone. Limnol Oceanogr 54: 132–144. doi:10.4319/lo.2009.54.1.0132.

