# Supplementary material for: Diversity and Spatial Distribution of Hydrazine Oxidoreductase (hzo) Gene in the Oxygen Minimum Zone Off Costa Rica
Source: PLoS One. 2013 Oct 31;8(10):e78275. doi: 10.1371/journal.pone.0078275 (PMC3814345; doi:10.1371/journal.pone.0078275)
Supplement: Figure S1 — Unifrac weighted PCoA of HZO community composition using the HZO cluster 1 and cluster 2 amino acid sequences. (DOC) [file pone.0078275.s001.doc]

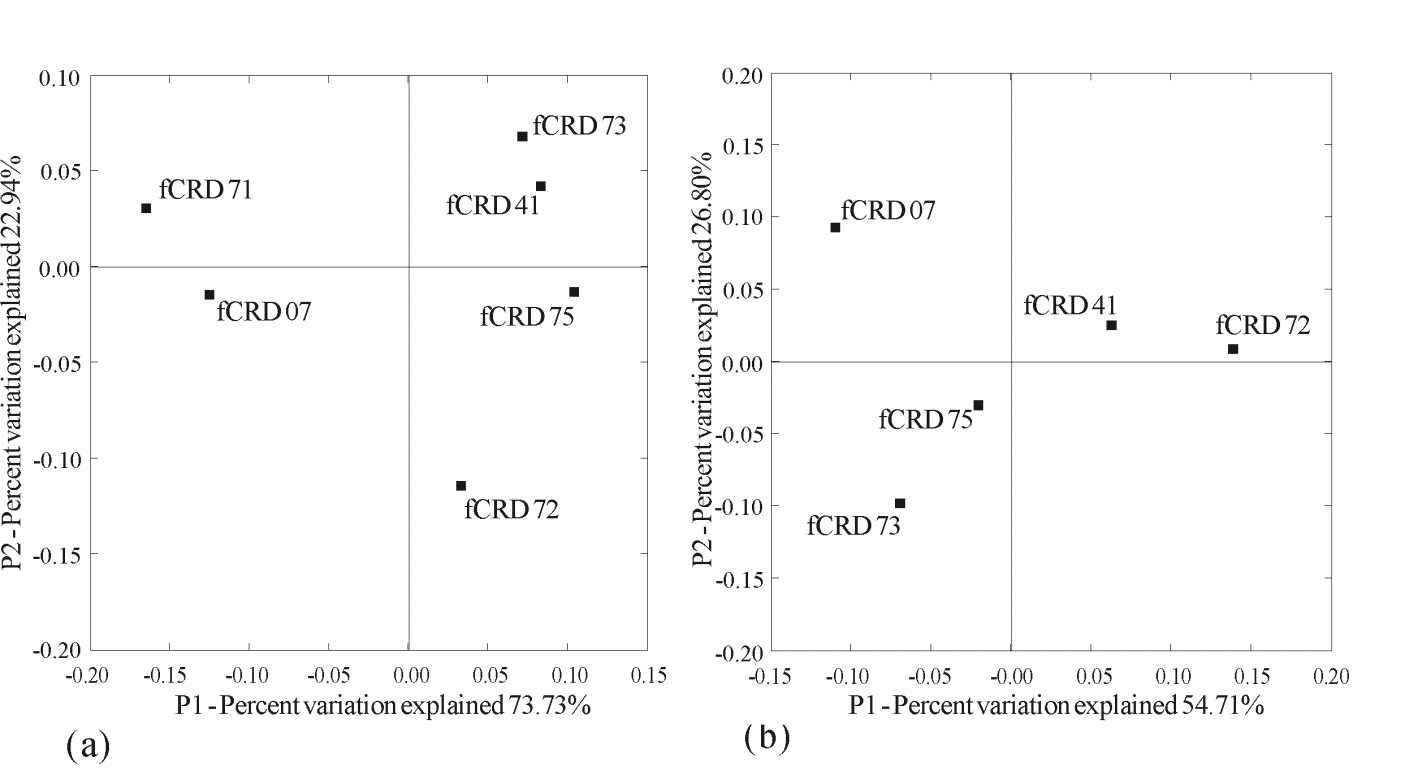


**Figure S1.** Unifrac weighted PCoA of HZO community composition using the HZO cluster 1 (a) and cluster 2 (b) amino acid sequences.
