# Supplementary figures and images for: Diversity and Spatial Distribution of Hydrazine Oxidoreductase (hzo) Gene in the Oxygen Minimum Zone Off Costa Rica
Source: PLoS One. 2013 Oct 31;8(10):e78275. doi: 10.1371/journal.pone.0078275 (PMC3814345; doi:10.1371/journal.pone.0078275)

**
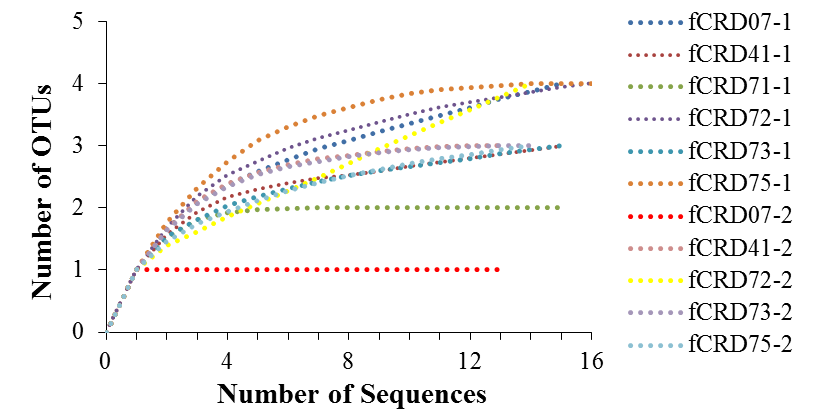
**

**Figure S2.** Rarefaction analysis of the six HZO cluster 1 and five HZO cluster 2 clone libraries.

Supplement: Figure S2 — Rarefaction analysis of the six HZO cluster 1 and five HZO cluster 2 clone libraries. (DOC) [file pone.0078275.s002.doc]
