# Supplementary material for: Diversity and Spatial Distribution of Hydrazine Oxidoreductase (hzo) Gene in the Oxygen Minimum Zone Off Costa Rica
Source: PLoS One. 2013 Oct 31;8(10):e78275. doi: 10.1371/journal.pone.0078275 (PMC3814345; doi:10.1371/journal.pone.0078275)
Supplement: Figure S3 — Neighbor-joining phylogenetic tree constructed with anammox bacteria 16S rRNA gene sequences from the oxygen minimum zone (OMZ) in Costa Rica Dome (CRD). (DOC) [file pone.0078275.s003.doc]

**
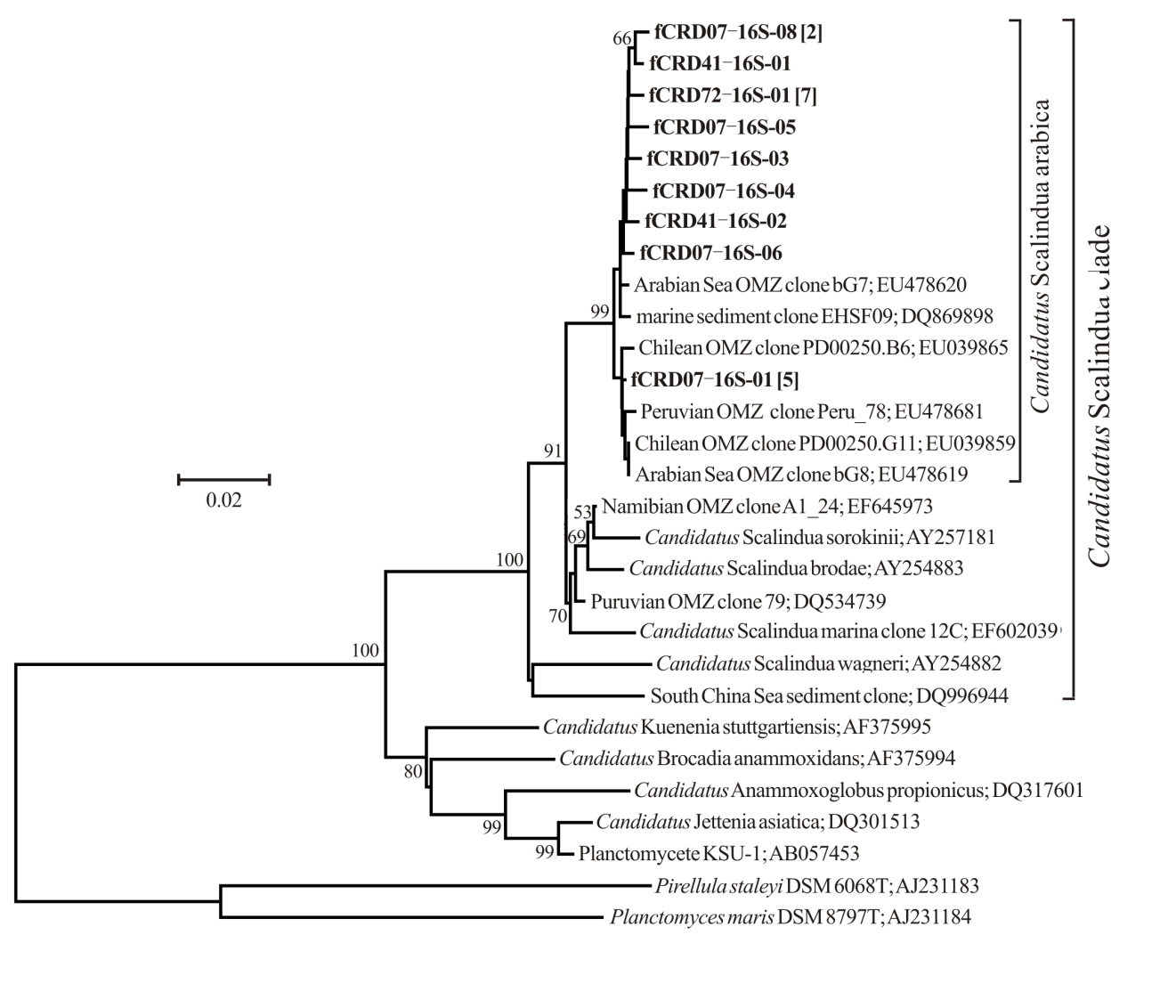
**

**Figure S3**. Neighbor-joining phylogenetic tree constructed with anammox bacteria 16S rRNA gene sequences from the oxygen minimum zone (OMZ) in Costa Rica Dome (CRD). The gene fragments were amplified with the primers Brod541F and Brod1260R (Penton *et al*., 2006).The sequences in bold were obtained in the present study. Bootstrap resampling was performed 1000 times and only its values higher than 50% are shown. *Pirellula staleyi* DSM 6069T and *Planctomyces maris* DSM 8797T were used as an outgroup.
