# Supplementary material for: Diversity and Spatial Distribution of Hydrazine Oxidoreductase (hzo) Gene in the Oxygen Minimum Zone Off Costa Rica
Source: PLoS One. 2013 Oct 31;8(10):e78275. doi: 10.1371/journal.pone.0078275 (PMC3814345; doi:10.1371/journal.pone.0078275)
Supplement: Figure S4 — Spearman's rho correlation of the hzo cluster 1 and cluster 2x sequences with nitrite concentration. (DOC) [file pone.0078275.s004.doc]

**
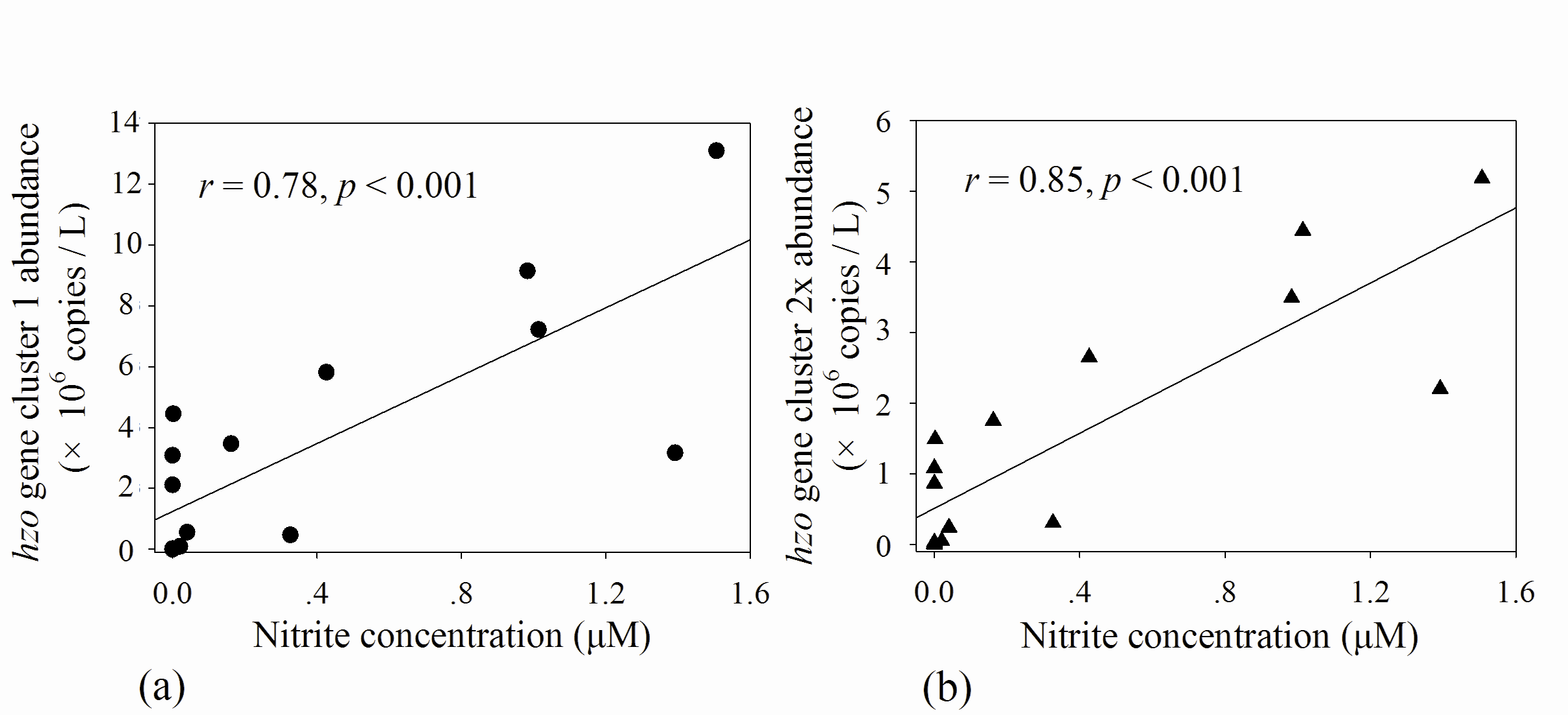
**

**Figure S4.** Spearman's rho correlation of the *hzo* cluster 1 (a) and cluster 2x (b) sequences with nitrite concentration.
