# Supplementary material for: Diversity and Spatial Distribution of Hydrazine Oxidoreductase (hzo) Gene in the Oxygen Minimum Zone Off Costa Rica
Source: PLoS One. 2013 Oct 31;8(10):e78275. doi: 10.1371/journal.pone.0078275 (PMC3814345; doi:10.1371/journal.pone.0078275)
Supplement: Figure S5 — Alignment of selected HZO cluster 1 and cluster 2x gene deduced amino acid sequences from this study and the representative HZO sequences from the Genbank. (DOC) [file pone.0078275.s005.doc]

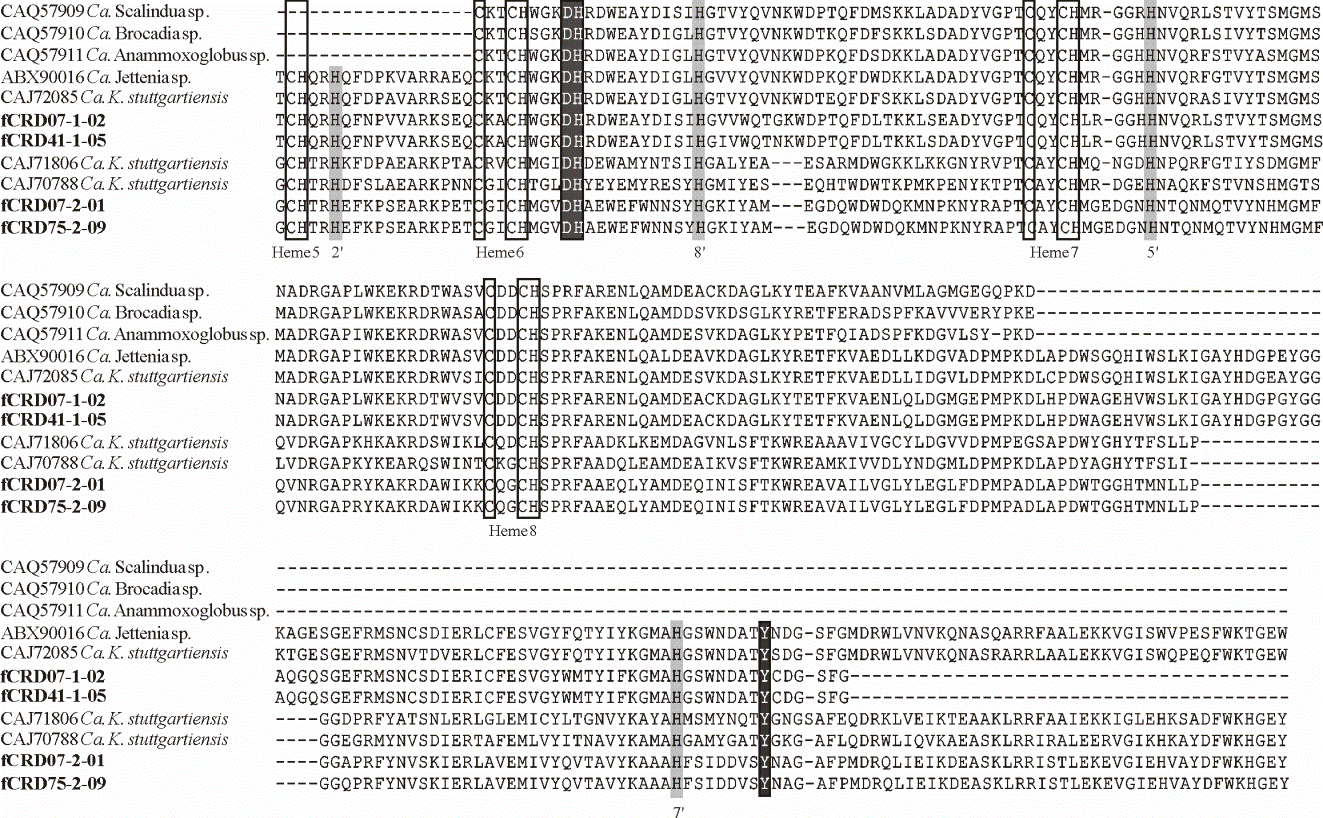


**Figure S5.** Alignment of gene deduced amino acid sequences of HZO cluster 1 (fCRD07-1-02, fCRD41-1-05), cluster 2x (fCRD07-2-01, fCRD75-2-09) and the representative HZO sequences from the Genbank. The gene sequences selected from this study for alignment were the representatives of the 2 most abundant OTUs in each cluster.
